# Association between first birth caesarean delivery and adverse maternal-perinatal outcomes in the second pregnancy: a registry-based study in Northern Tanzania

**DOI:** 10.1186/s12884-022-04719-7

**Published:** 2022-05-16

**Authors:** Raziya Gaffur, Bariki Mchome, Lyasimana Lithaneninn Ndaninginan, Benjamin Asubiojo, Michael Johnson Mahande, Eusebious Maro

**Affiliations:** 1 Kilimanjaro Christian Medical University College, P.O Box 2240, Moshi, Tanzania; 2 Department of Obstetrics and Gynecology, Kilimanjaro Christian Medical Center, P.O Box 3010, Moshi, Tanzania; 3 Department of Epidemiology and Biostastics, Institute of Public Health, Kilimanjaro Christian Medical University College, P.O Box 2240, Moshi, Tanzania

**Keywords:** Caesarean delivery, Maternal -Perinatal outcomes, Tanzania

## Abstract

**Background:**

Caesarean delivery (CD) is the commonest obstetric surgery and surgical intervention to save lives of the mother and/or the new-borns. Despite been accepted as safe procedure, caesarean delivery has an increased risk of adverse maternal and fetal outcomes. The rising rate of caesarean delivery has been a major public health concern worldwide and the consequences that come along with it urgently need to be assessed, especially in resource limited settings. We aimed to examine the relationship between first birth caesarean delivery and adverse maternal and perinatal outcomes in the second pregnancy among women who delivered at a tertiary hospital in Northern Tanzania.

**Methods:**

A retrospective cohort study was conducted using maternally-linked data from Kilimanjaro Christian Medical Centre. All women who had singleton second delivery between the years 2011 to 2015 were studied. A total of 5,984 women with singleton second delivery were analysed. Multivariable log-binomial regression was used to determine the association between first caesarean delivery and maternal-perinatal outcomes in the second pregnancy.

**Results:**

Caesarean delivery in the first birth was associated with an increased risk of adverse maternal and perinatal outcomes in the second pregnancy. These included repeated CD (ARR 1.19; 95% CI: 1.05–1.34), pre/eclampsia (ARR 1.38; 95% CI: 1.06–1.78), gestational diabetes mellitus (ARR 2.80; 95% CI: 1.07–7.36), uterine rupture (ARR 1.56; CI: 1.05–2.32), peri-partum hysterectomy (ARR 2.28; CI: 1.04–5.02) and preterm birth (ARR 1.21; CI: 1.05–1.38).

**Conclusion:**

Caesarean delivery in their first pregnancy had an increased risk of repeated caesarean delivery and other adverse maternal-perinatal outcomes in the following pregnancy. Findings from this study highlight the importance of devising regional specific measures to mitigate unnecessary primary caesarean delivery. Additionally, these findings may help both clinicians and women in deciding against or for trial of labor after previous caesarean delivery in an event of absent direct obstetric indication.

**Supplementary Information:**

The online version contains supplementary material available at 10.1186/s12884-022-04719-7.

## Introduction

Globally, the rising rate of caesarean delivery (CD) has been a major public health concern to the public health worldwide [1]. There are wide variations in respect to the management of pregnancy in women with previous caesarean delivery and more specifically CD in the first birth between high-income and low-income countries [2]. Over the three decades, evidence showed a worldwide spiked rate of CD ranging from 6–40% [3], and primary CD being the most common indicator among women with previous CD [4, 5]. Most recent CD rate in Tanzania was estimated to be 6%, with an estimated rate of 11% for Kilimanjaro region while the CD rate at KCMC hospital, a tertiary hospital for Northern zone of Tanzania is reported ranging between 29.9–35.5% [6–8].

In 2015, two thirds of the global maternal deaths were reported to occur in Sub-Saharan Africa (SSA), and the maternal mortality ratio was estimated to be twice the global average of 546 per 100,000 live births [9]. The CD rate in SSA has been stagnant at 3.5% compared to the increasing CD rate globally, yet the maternal death after CD is fifty times higher compared to high income countries [9].

A major challenge is that previous CD is one of the leading indication for the repeated caesarean delivery in the subsequent delivery [10]. Inadequate counselling offered by physicians and midwives to women who had previous one CD might have been attributed to the increased rate of repeat CD. Indeed, many of these women report in labor with limited or no knowledge regarding the possibility of a trial of labor [10]. In addition to this, inability to adequately monitor the fetus and safely augment the progress of labor when the choice is to proceed with a trial of labor is challenging [10, 11].

Several studies conducted in developing countries in Europe, Asia and America have demonstrated the complex association of first CD with maternal and fetal morbidity and mortality in the second pregnancy. These include increased risks of repeated CD, pre-eclampsia, placenta previa, placenta abruption, post-partum haemorrhage, uterine rupture, peri-partum hysterectomy, preterm birth, unexplained antepartum fetal death, and low birth weight [3, 12–18]. However, in most sub-Saharan Africa countries including Tanzania, where limited resources coupled with a relatively higher maternal mortality ratio is overwhelming, the extensiveness of this association has not much been widely studied. Additionally, with the increasing rate of CD in these settings, it is imperative to study the risks of the first CD which can then be communicated to the patient for their future reproductive planning. This study aimed to examine the association between first birth CD and maternal-perinatal outcomes in the second pregnancy among women who delivered at Kilimanjaro Christian Medical Centre a tertiary health hospital in Northern Tanzania.

## Materials and methods

### Study design and setting

A medical birth registry-based retrospective cohort study was designed using maternally-linked data from Kilimanjaro Christian Medical Centre (KCMC). A five years’ data from 1^st^ January, 2011 to 31^st^ December 2015 was reviewed. KCMC hospital tertiary referral hospitals and a university teaching hospital located in the Northern zone of Tanzania. The hospital receives women from the local community and referrals from the nearby regions including Arusha, Manyara, Tanga, and Singida. The average number of deliveries per year is between 4000–4800 deliveries. Over the period of 6 years (2005–2010), the rate of CD at KCMC is 29.9–35% [7].

### Data source and data collection

The KCMC medical birth registry was established in 2000 to serve clinical, administrative and research purposes and to date, over 70,000 births have been recorded. All women who delivered at KCMC undergo a prospective interview with a standardized questionnaire within 24-h of delivery or later in case of any delivery complications. The interview is conducted by the trained midwives at the department of Obstetrics and Gynaecology. The details of the interview procedures has been described elsewhere [19]. Information regarding birth outcomes, delivery mode, obstetric history and socio-demographic is recorded in the birth registry, including information of neonates admitted to neonatal care unit. For women who delivered their first birth at KCMC, we linked the mother’s record with the child’s by unique number, which is assigned to every woman who delivers at KCMC.

### Study population

**Fig. 1:**
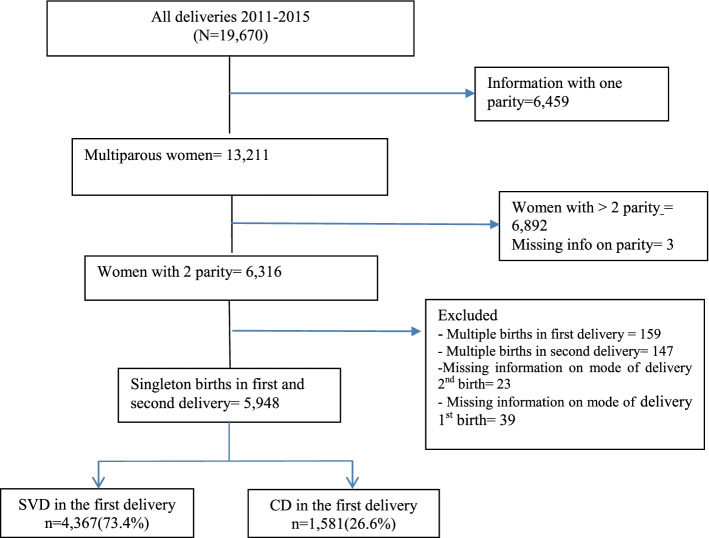
Flow diagram for participants’ selection (sample size estimation)

Data for 19,670 women who delivered within the years 2011 to 2015 were obtained from KCMC birth registry. Of these, 13,211 women were multiparous. The study sample included only data for women with complete information of the second singleton delivery within the study period, and whose records were available at KCMC medical birth registry. These women were further classified into two groups; those with first birth CD and those with spontaneous vaginal delivery (SVD). Women with missing information on the mode of delivery in their first and second pregnancies were excluded. The total of 13,722 women were excluded according to our exclusion criteria. The remaining, 5,948 women had singleton deliveries in the second birth, of which 4,367(73.4%) and 1,581(26.6%) women had SVD and CD respectively in their first birth, therefore they constituted the final sample size and were analysed (Fig. 1).

### Study variables and definitions

#### Dependent variable

The primary outcome variables in this study were adverse maternal and perinatal outcomes in the second pregnancy. Adverse maternal outcomes included caesarean delivery, Abruption placenta, Placenta Previa, Postpartum haemorrhage, Pre/eclampsia, Gestational Diabetes Mellitus, Uterine Rupture and Peri-partum- Hysterectomy. Adverse perinatal outcomes included Apgar score less than 7 at 5^th^ minutes, admission to a neonatal care unit, low birth weight (LBW), fetal macrosomia, preterm birth, still birth and neonatal death before 24 h.

#### Independent variables

The main exposure of interest was first birth caesarean delivery. It was defined as caesarean delivery preformed during the first pregnancy for the first time. Other covariates included maternal age which originally were recorded as continuous and then was categorized into 15–19 years, 20–29 years, 30–39 years and ≥ 40 years, maternal education, Body Mass Index (BMI) was calculated as ante-natal care booking body weight in kg/ height in metres squared, then was categorized according to the World Health Organization (WHO) standard; Normal weight 18.5–24.9 kg/m^2^_,_ underweight < 18.5 kg/m^2^, overweight 25.0–29.9 kg/m^2^ and obese ≥ 30 kg/m^2^. Gestational age at delivery was recorded in weeks as continuous, and then was categorized as 28-32 weeks, 33–36 weeks and ≥ 37 weeks. Inter-pregnancy interval was calculated from year of delivery in the first pregnancy to the year of second pregnancy which was recorded as continuous, and then was converted into months: ˂ 24 months, 24–36 months, 37–60 months and ˃ 60 months. Referral status was divided into three categories: home, district hospital and regional hospital. Obstetric outcomes in first birth such as Pre/eclampsia, Epilepsy, GDM, anaemia, heart disease, still birth, macrosomia, low birth weight and preterm birth were assessed in both groups.

### Statistical analysis

Data analysis was performed using STATA version 13.0 (StataCorp.2013.College Station, TX: StataCorp LP). Descriptive statistics were summarized using mean and standard deviation for continuous variables while frequency and proportion was used to summarize the categorical variables. The chi-square test was used to determine the association between the first birth CD with baseline maternal and obstetric characteristics in bivariate analysis. Both crude relative risk (CRR) and adjusted relative risk (ARR) with 95% confidence intervals for the association between the first birth CD and maternal-perinatal outcomes in the second delivery wa estimated using multivariable log-binomial regression model. A *p*-value of < 0.05 was considered statistically significant.

### Ethical considerations

The study was approved the Kilimanjaro Christian Medical University College Research and Ethics Committee (ethical clearance certificate number: 2346). Permission to use the medical birth registry data was obtained from the KCMC hospital administration.

## Results

### Characteristics of the study participants

The demographic characteristics of the study participants are shown in Table 1. The mean age among both groups was 28.3 (SD = 5.4) years. The women with first birth CD, 653 (41.3%) were overweight and in respect to gestation age at time of delivery, both grouped women delivered at term 3408(78.0%) and 1187(75.1%) for SVD and CD respectively. The inter-pregnancy interval ranged between 37–60 months among women with CD (62.4%) in the first birth which was higher compared to SVD (55.5%) group and had significant difference.

**Table 1:** Baseline characteristics of the study participants with singleton birth in the second pregnancy (*N* = 5948)

**Characteristics**	**Women with first birth SVD (*n* = 4367)**		**Women with first birth CD (*n* = 1581)**		***P*-value**
	**n**	**%**	**n**	**%**	
**Age [Mean, SD]**	**[28.3, 5.4]**		**[28.3, 5.4]**		
**Maternal Age**					
15–19	163	3.7	63	4.0	0.822
20–29	2483	56.9	911	57.6	
30–39	1617	37.0	574	36.3	
≥40	104	2.4	33	2.1	
**Education**					
None formal	64	1.5	27	1.7	0.685
Primary education	1844	42.2	689	43.6	
Secondary education	1126	25.8	398	25.2	
Higher education	1333	30.5	467	29.5	
**Religion**					
Christian	3476	79.6	1243	78.6	0.604
Muslim	865	19.8	326	20.6	
Others	26	0.6	12	0.8	
**Occupation**					
Housewife	517	11.8	186	11.8	0.444
Farmer	650	14.9	209	13.2	
Business	1454	33.3	554	35.0	
Employed	1376	31.5	490	31.0	
Others e.g. students, retired	370	8.5	142	9.0	
**Residency**					
Urban	2584	59.2	937	59.3	0.947
Rural	1783	40.8	644	40.7	
**Tribe**					
Chagga	2242	51.3	793	50.2	0.857
Pare	527	12.1	200	12.7	
Maasai	93	2.1	35	2.2	
Others	1505	34.5	553	35.0	
**Booking BMI (kg/m**^**2**^**)**					
Underweight	131	3.0	70	4.4	** < 0.0001**
Normal	2058	47.1	649	41.0	
Overweight	1762	40.3	653	41.3	
Obesity	416	9.5	209	13.2	
**[Mean, SD]**	**[24.7, 3.5]**		**[25.3, 4.4]**		
**ANC visit**					
≥4	2882	66.0	1057	66.9	0.535
4	1485	34.0	524	33.1	
**Gestation age at delivery**					
28–32	130	3.0	49	3.1	**0.047**
33–36	829	19.0	345	21.8	
≥37	3408	78.0	1187	75.1	
**[Mean, SD]**	**[37.9, 2.3]**		**[37.8, 2.3]**		
**Inter-pregnancy Interval**					
< 24 months	15	0.3	7	0.4	** < 0.0001**
24–36 months	1200	27.5	338	21.4	
37–60 months	2423	55.5	987	62.4	
> 60 months	729	16.7	249	15.7	
**Referral**					
Home	3508	80.3	1227	77.6	0.566
District hospital	707	16.2	289	18.3	
Regional hospital	152	3.5	65	4.1	

### First birth obstetric characteristic of study participants

Table 2 displays the obstetric characteristics of the first birth among women with CD and SVD. Both grouped women delivered at term (73.1% vs. 68.8%, for those with CD and SVD in their first pregnancy) respectively. Women with CD were more likely to have Pre/eclampsia and GDM [(5.5% vs. 4.0% and (0.8% vs. 0.3%)] respectively, in the first birth than those with SVD, although it was of no significance. In respect to the fetal characteristics, women with first birth CD were more likely to have macrosomia (11.0% vs.7.5%, *P* < 0.0001) than the contrast group. However, the women with first birth CD were less likely to have preterm birth and stillbirths [(5.1% vs. 3.4%) and (29.0% versus 25.4%)], respectively than those with SVD.

**Table 2:** First birth obstetric characteristic of study participants (*N* = 5948)

Characteristics	Women with first birth SVD (*n* = 4367)		Women with first birth CD (*n* = 1581)		*P*-value
	**n**	**%**	**n**	**%**	
**Maternal Characteristics**					
**Gestation age**					
28–32	95	2.2	25	1.6	0.005
33–36	1269	29.1	401	25.4	
≥37	3003	68.8	1155	73.1	
**Medical Conditions**					
**Pre/Eclampsia**					
Yes	173	4.0	87	5.5	0.010
No	4194	96.0	1494	94.5	
**Epilepsy**					
Yes	37	0.8	11	0.7	0.564
No	4330	99.2	1570	99.3	
**GDM**					
Yes	11	0.3	13	0.8	0.002
No	4356	99.7	1568	99.2	
**Anaemia**					
Yes	48	1.1	20	1.3	0.595
No	4319	98.9	1561	98.7	
**Heart Disease**					
Yes	47	1.1	20	1.3	0.542
No	4320	98.9	1561	98.7	
**Fetal characteristics**					
**Still birth**					
Yes	222	5.1	54	3.4	0.007
No	4145	94.9	1527	96.6	
**Macrosomia**^**a**^					
Yes	326	7.5	174	11.0	< 0.0001
No	4041	92.5	1407	89.0	
**Low birth weight**^b^					
Yes	371	8.5	138	8.7	0.776
No	3996	91.5	1443	91.3	
**Preterm birth**					
Yes	1268	29.0	401	25.4	0.005
No	3099	71.0	1180	74.6	

^a^Birth weight ≥ 4000 g; ^b^Birth weight ≤ 2500 g

### Association between first birth caesarean delivery and adverse maternal outcomes in the second pregnancy

The relationship between first birth caesarean delivery and adverse maternal outcomes has been displayed in Table 3. Women with first birth CD had an increased risk of having CD [ARR1.19 (95% CI: 1.05–1.34)]; pre/eclampsia [ARR1.38(95% CI: 1.06–1.78)], GDM [ARR 2.80 (95% CI: 1.07–7.36)], uterine rupture [ARR1.56 (95%CI: 1.05–2,32)] and peri-partum hysterectomy [ARR2.28 (95% CI: 1.04–5.02)] in their second pregnancy as compared to their counterparts who had SVD. The association between CD in the first pregnancy and placenta abruption, placenta previa, and PPH was not statistically significant (Table 3).

**Table 3:** Association between first birth caesarean delivery and adverse maternal outcomes in the second pregnancy (*N* = 5948)

Maternal outcomes	Women with first birth SVD^c^ (*n* = 4367)	Women with first birth CD (*n* = 1581)	CRR (95% CI)	ARR (95% CI)
Cesarean delivery	1568(35.9)	648(41.0)	1.24(1.10–1.39)	1.19(1.05–1.34)^**a**^
Placental abruption	73(1.7)	32(2.0)	1.22(0.80–1.85)	
Placental previa	80(1.8)	37(2.3)	1.28(0.87–1.90)	
PPH	291(6.7)	122(7.7)	1.17(0.94–1.45)	
Pre/eclampsia	207(4.7)	109(6.9)	1.49(1.17–1.89)	1.38(1.06–1.78)^**b**^
GDM	15(0.3)	14(0.9)	2.59(1.24–5.38)	2.80(1.07–7.36)^**b**^
Uterine rupture	82(1.9)	43(2.7)	1.46(1.01–2.12)	1.56(1.05–2.32)^**b**^
Peripartum hysterectomy	18(0.4)	15(0.9)	2.31(1.16–4.60)	2.28(1.04–5.02)^**a**^

*CRR* Crude Relative Risk, *ARR* Adjusted Relative Risk, *CI* Confidence Interval

^a^Adjusted by Maternal age, Booking BMI, Gestation age, Inter-pregnancy interval, also current and previous pre/eclampsia and GDM

^b^adjusted by Maternal age, Gestational age, Booking BMI, Inter-pregnancy interval, previous GDM, and pre/eclampsia

^c^Reference group

### Association between first birth caesarean delivery and adverse perinatal outcomes in the second pregnancy

In unadjusted analysis, having first CD was associated with preterm birth in the second pregnancy [CRR1.18 (95% CI: 1.03–1.34)]. In multivariable model, the association between first birth CD in the first pregnancy and preterm birth in the second pregnancy also remained significant [ARR1.21 (95% CI: 1.05–1.38)]. However, there were no significant difference in terms in low birth weight (LBW), macrosomia, low Apgar sore in 5^th^ minute, still birth and early neonatal death between women with previous CD and those without (Table 4).

**Table 4:** Association between first birth caesarean delivery and adverse perinatal outcomes in the second pregnancy (*N* = 5948)

Adverse outcomes	^h^Women with first birth SVD (*n* = 4367	Women with first birth CD (*n* = 1581)	CRR(95%CI)	ARR(95%CI)
Preterm birth	959(22.0)	394(24.9)	1.18(1.03–1.34)	1.21(1.05–1.38)^a^
Neonatal unit admission	650(14.9)	219(13.9)	0.92(0.77–1.08)	0.90(0.74–1.09)^b^
Macrosomia	257(5.9)	72(4.6)	0.76(0.58–0.99)	0.72(0.55–0.95)^c^
Low birth weight	417(9.5)	165(10.4)	1.10(0.91–1.33)	0.96(0.78–1.18)^d^
Low Apgar score in 5 min (*n* = 5771)	89(2.1)	36(2.3)	1.12(0.75–1.65)	1.06(0.71–1.58)^e^
Still birth	128(2.9)	49(3.1)	1.06(0.75–1.47)	0.95(0.67–1.36)^f^
Neonatal death in 24 h	35(0.8)	20(1.3)	1.58(0.91–2.75)	1.72(0.73–4.03)^g^

*CRR* Crude Relative Risk, *ARR* Adjusted Relative Risk, *CI* Confidence Interval

^a^adjusted by the current preeclampsia GDM Macrosomia, LBW, and previous preterm

^b^adjust by Preeclampsia, GDM, Macrosomia, LBW, Apgar score in 5 and Preterm

^c^adjusted by macrosomia in the first birth, pre/eclampsia, GDM, stillbirth, and preterm in the second birth

^d^adjusted by LBW in the first birth, pre/eclampsia, GDM, and Preterm in the second birth

^e^adjusted by preeclampsia, GDM, LBW, Preterm, abruption placenta in the second birth

^f^adjusted by stillbirth in the first birth, preeclampsia, GDM,, Macrosomia, Preterm and abruption placenta in the second birth

^g^adjusted by preeclampsia, GDM, macrosomia, LBW, Apgar score in 5 Preterm, and Abruption placenta in the second birth

^h^Reference group

## Discussion

In the present study, CD in the first pregnancy was associated with higher risk of adverse maternal and perinatal outcomes in the second pregnancy. We found that women with initial CD had nearly two fold increased risk of repeated CD in their second pregnancy.

The finding in our study is consistent with previous studies done in China and Germany [3, 12]. The reason for high repeated CD for example in China was due to ‘two child policy’, leading to increase in maternal request for CD, to get the next precious baby. In the present study, the high repeated CD could be explained by the nature of the studied population and being conducted at the tertiary hospital that receives pregnant women in different state and conditions of labor, at which to perform CD maybe best possible form of mode of delivery for the attending physician. In addition to this the dilemma and pressure on the doctors is fact that the physician may have not attended to the patient prior and also inadequate information on the first CD before the labor.

In consistent with other studies, this study revealed that first birth CD is associated with significant increased risk of adverse outcomes: pre/eclampsia, GDM, uterine rupture and peri-partum hysterectomy. Supporting the finding to this study, specifically on pre/eclampsia, study in USA and Peru has shown three folds high and almost one half high risk respectively in the subsequent pregnancy [14, 20]. Hu et al. had contrary to this evidence, did not identify any risk in this relationship [3]. Although the mechanism underlying this association is unclear, the most likely explanation is that cesarean section scar leads to change in the endometrium; hence the pathophysiology for pre/eclampsia is supported by poor trophoblast invasion, less vascularization and incomplete remodelling of spiral arteries [21, 22]. However, in our study we could not do subgroup analysis in respect to indications of cesarean delivery especially for the first delivery and difference in race and ethnicity as incidence of pre/eclampsia is higher in African-American women [23]. Another finding is the increased risk of uterine rupture in the second pregnancy in women with first birth CD. Worth mentioning, is higher number of uterine ruptures with limited peri-partum hysterectomy. This disparity is possibly due to repairs of some uterine rupture which were not captured in the registry [13, 14]. Despite uterine scar being a major risk factor for uterine rupture, unscarred uterus may similarly present with uterine rupture as observed in the current study. Previous studies have documented similar findings [24, 25].

Furthermore, this study also found association of first birth CD and increased risk of developing GDM and peri-parturm hysterectomy in the second pregnancy. Although this association is statically significant, the explanation that could account for this, especially for GDM is the small number of events, thus wide confidence interval. Conversely, Hu et al. in China had similar association with larger events [3]. Similar to pre/eclampsia, GDM could be associated with increasing placenta mass which directly influenced anti-insulin hormones production [23].

In relation to maternal outcomes, our findings show that there were some association between first birth CD and placenta abruption, placenta previa and PPH, however not significant, which is consistent with other analysis in respect to placenta previa and PPH [13], however contrary to Hu et al. and Kennare et al. [3, 16]. Possible explanation that may have influenced our finding might be missing information on complication in the first birth which may have influenced the current pregnancy and furthermore our data did not classify the types of placenta previa.

On the aspect of perinatal outcomes, we found that, women with first birth CD had 1.21(CI 95%: 1.05–1.38) higher risk of having preterm birth in the second pregnancy similar to a systemic review and meta-analysis, which involved ten retrospective cohort studies with more than ten million women, showing CD in the first pregnancy increased risk of preterm birth (ARR 1.12, 95%CI 1.01–1.24) in the subsequent pregnancies [26]. This association is explained by the changes in the intra-uterine structure and its microenvironment, although the pathogenesis of this event is unclear [26]. However, inability to control for other possible confounders such as premature rupture of membranes, infections or cigarette smoking which could lead to preterm delivery was a limitation that could have given different view to our finding. Also the recorded preterm birth was not categorized as either induced or spontaneous and on the other hand, availability of neonatal care facility at KCMC could influence the results in our study, as most of women with preterm labour are referred to KCMC for preterm delivery and neonatal care.

### Strengths and limitations

Our study included data for five years; hence large scale sample size. Our analysis was strictly restricted to women with first and second singleton pregnancies, thereby eliminating potential confounding effect of parity and multiple gestation pregnancy. Being a secondary data from birth registry, we could not capture many important factors that could have helped us in better analysis, such as indications of previous and current cesarean delivery, history of myomectomy, history of previous placental abruption, where was the first cesarean delivery performed, inability to capture the women with GDM who were tested for OGTT, possibility of leading to inappropriate documentation of certain clinical condition, such as birth-weight, gestational age. These findings may not reflect the situation in the other settings and population in whole, as KCMC being a tertiary hospital, may be leading to referral bias and information retrieved from birth registry as a secondary data may be limited in its completeness. A multi-centred prospective data collection may enrich the quality of information and true reflection of the findings.

## Conclusion and recommendation

In view of our findings, caesarean delivery in the first birth appears to increase the risk of a repeated caesarean delivery and other adverse outcomes in the second pregnancy. We emphasise clinicians to try to balance the risks and benefits of caesarean delivery in the first and future births. However, we should ensure adequate counselling of the women during ante-natal visits on risks versus benefits of and caesarean delivery and vaginal birth after caesarean delivery.

## Supplementary Information

**Additional file 1. **

## Data Availability

The datasets generated during and/or analysed during the current study are available from the corresponding author on reasonable request.

## Abbreviations

ANC: Ante-natal care
BMI: Body Mass Index
CD: Caesarean delivery
CI: Confidence Interval
GDM: Gestational Diabetes Mellitus
KCMC: Kilimanjaro Christian Medical Centre
LBW: Low birth weight
PPH: Post-partum Haemorrhage
SSA: Sub-Saharan Africa
SVD: Spontaneous vaginal delivery
