# Supplementary material for: Association between first birth caesarean delivery and adverse maternal-perinatal outcomes in the second pregnancy: a registry-based study in Northern Tanzania
Source: BMC Pregnancy Childbirth. 2022 May 16;22:411. doi: 10.1186/s12884-022-04719-7 (PMC9112465; doi:10.1186/s12884-022-04719-7)
Supplement: Supplementary file 1 — Additional file 1. [file 12884_2022_4719_MOESM1_ESM.pdf]

Association between first birth caesarean delivery and adverse maternal-perinatal outcomes in the second pregnancy: a registry-based study in Northern Tanzania

Raziya Gaffur, Bariki Mchome , Lyasimana Lithaneninn Ndaninginan, Benjamin Asubiojo , Michael Johnson Mahande and Eusebious Maro.

KCMC Medical Birth Registry

Version 5 - November 2004

1 Basic information conceFf4ing mc›ther

1.1 Mothers date of birth . , Age:
-* Mothers name :
1.4 Hospital number:
1.3 Address:
1.6 Birth number:
1.S Date of interview:
1.8 Date of admission:
1.7 Intewiew by:

Referred for delivery: 1 Yes if yes: zzpReferred from:
z No (Set referral
1 Home
z Regional hospital
3 District hospital
4 ther, specify:

Reason for referral:

Referred during labour:
1 Admitted in labour
2 Admitted before labour

1<9 Official date of discha rge:(
1.10 Date leaving **hospital:**

1.11 Current residence:
1 Rural
I Urban
3 Semi urban

1.12 Mothers childhood residence:
Rural
2 Urban
› Semi urban

Area of mother.s residence:
Area of mother's childh0Od residence:

1.13 Highest educational levet:
None
2 Pfimary (1-7)
Secondary t8-11)
4 Higher (12+)

1.14 Current occupation:
1 HOusewife
2 Farmer
z Service
4 Business
s ProTessona|
ustudent
Others ⇒

1.1S Current marital status:
Married Age at first marriage:
z Single
3 Wiaowed
‹ Remarried
5 Divorced
s Polygamous family Add wife number:

No of previous pregnancies

1.16 Regular menstrual periods:
Yes
2 g o
Age at menarche:

1.17 Genital mutilation {C/rcomc/s/on}
1 Yes
Q z No
If yes, at age:
If yes, type:
Type one
2 Type two
* Type three
4 Other types

1.18 Mother's tribe:
0\ Chagga
z Pane
03 Masai
Other

1.19 Religion:
1 Catholic
2 Protestant
s Muslim
^ Others =>

2 Questions concern ng the father of the child:

2.1 Father's name:
- Father*s age:

2.3 Current occupation of father:
z1 Farmer
0z Business
33Skilled worker
•‹ Unskilled wOrker'
as Servise
06 Official
cz Professional
06 Student
09 Unemployed
10 Other II

2.4 Father's educational level:
I None
z Primary (1-7)
z Secondary {8-11)
4 Higher t12+)

2.5 Father's tribe:
‹ Chagga
2 Pare
z Masai
^ Others »

3 Questions concerning home conditions:

3.1 Source of drinking water:
1 Tap water
2 We/i
3 River
4 Spring

3.3 Qj stance to water, if not tap:
1 Less than 1 km (less hour walk)
z More than 1 km, specify in km:

3.4 }-{ome toilet:
Pit latrine

4 Mothers health before and during present pregnancy

4.1 Body weight (kg): (before pregnancy) [ ]

4.2 Body height (cm): [ ]

4.3B lood transfusions: 1 Yes / › No

4.4 Serious diseases
- [ ] O1 Diabetes
- [ ] Hypertension
- [ ] o3 Heart diseases
- [ ] 04 Epilepsy
- [ ] os Malaria
- [ ] 06 Anaemia
- [ ] oz Gynaecological disease
- [ ] os Liver disease (jaundice)
- cs k idney disease
- [ ] i9 Lung disease
- [ ] -1 Tuberculosis
- 12 Sickle celt
- 13 Other, sp ecifv t/ [ ]

4.5 Have you ever practised family planning:
- [ ] 1 Yes
- [ ] 2 No

Months trying to get pregnant: [ ]

If yes, y/hat kind Of prevention
- [ ] 01 Pills
- [ ] z Injections
- [ ] C3 I IN D
- [ ] ›• Condoms
- [ ] o5 [mplant
- [ ] 06 Lactation
- [ ] 07 Withdrawal
- [ ] ca Matural
- 09 Abstinence
- [ ] 1o Traditional
- [ ] 11 Other specify

4.6 Antenatal care in this pregnancy:
- [ ] 1 Yes
- [ ] z No

i tye p; First medical appointment date: [ ]

Number of visits: [ ]

If date unknown, estimate first appointment :
- 1 0-12. week of gestation
- [ ] 2 13-20. week
- [ ] 3 21 30. week
- [ ] 4 After 31. week

4.7 L.M.P: [ ]

4.B Ultrasound
- [ ] › Yes
- [ ] No

4.9 E.D.D. Based on clinical estimate: [ ]

4M0 Do you smoke?
- [ ] 1 Yes
- [ ] z No

If yes: how many cigarettes per day: [,]

Smoking during this pregnancy:
- [ ] 1 Ycs
- [ ] 2 @O

Chewing tobacco
- [ ] 1 Yes
- [ ] 2 No

Chewing tobacco during this pregnancy:
- [ ] 1 Yes
- [ ] 2 No

› 11 Do you drink alcoholic beverages?
- [ ] 1 Yes
- [ ] z No

If yes
- [ ] Every day
- [ ] 2 More than once a week
- [ ] 3 Once a week
- [ ] 4 Occasionally

Did you also drink alcoholic beverages during this pregnancy?
- [ ] Yes
- [ ] 2 No

If yes:
- [ ] Every day
- [ ] 2 More than once a week
- [ ] 3 Once a week
- [ ] 4 Occasionally

4.12 Drugs on regular basis?
- [ ] 1 Yes W [ ]
- [ ] 2 No

If yes:
- [ ] 1 MOdern
- [ ] 2 Traditional

Did you take any drugs during this pregnancy:
- [ ] 1 Yes [ ]
- [ ] 2 No

If yes, specify:
- [ ] 1 Modern
- [ ] 2 Traditional

Did you take any drugs at time of conception or during first trimester:
- [ ] 1 Yes W [ ]
- 2 No

Drugs for infertility:
- [ ] Yes [ ]
- [ ] 2 No

4.15 Blood group (Ab0) [ ]

Rh: [ ]

Anti-D in previous pregnancies: 1 Yes / › No

VDRL status
- [ ] 1 Positive
- 2 Negative
- [ ] 3 Unknown

Hb [ ]

Hb measurement done:
- [ ] 1 On Admission
- [ ] 2 Last visit to ANC
- [ ] a Unknown

I•IIV test recorded
- [ ] › yes
- [ ] • No

If yes, result:.
- [ ] 1 negative
- z Positive

Treatment during this pregnancy:
- [ ] 1 YES
- [ ] 2 No

Spec|y [ ]

4.t6 Diseases and complications during present pregnancy, including accidents:
- [ ] Yes (specify below)
- [ ] 2 No
- [ ] *' Gestational diabetes
- [ ] •° Diabetes
- [ ] oz Hypertension
- [ ] Preeclampsia, mild
- [ ] *J Preeclampsia, severe
- [ ] as Eclampsia
- [ ] •‹ Epilepsy
- [ ] c/ Bleeoinq
- or Anaemia
- [ ] og Hyperemesis
- [ ] 1o Malaria
- [ ] 11 Jaundice
- [ ] 2Schistosomiasis
- [ ] 1a Gynaecological disease
- 14 Trom boem frolic disease
- [ ] 1s Heart disease
- ‹6 Tuberculosis
- [ ] 17 Lung disease
- ‹s Infectfons, specify
- [ ] 19 Others, specify

fl Questions concerning the delivery

-5 1 At birth ☐ 1 Single birth ☐ 2 Multiple births — If multiple, add ı"ıO. of children: ☐

Weight on admission: ☐

5.2 Complications during delivery
☐ 1 PROM
☐ z Bleeding > 500 mi
☐ 3 3-4. degree tear
☐ • Abruption of placenta
☐ s Placenta previa
☐ Other complications

Specify type other type of complication

5.3 Induction of labouF ☐ Yes ☐ 2 No

If yes: ☐ 1 Amniotomy ☐ z Oxytocin ☐ Prostaglandin

5.4 Others: 1 Episotomy ☐ 2 Symphysiotomy

5.5 Analgesia: 1 Yes ☐ z No

s.s Blood Loss **(ml)** ☐

5.6 Anaesthesia: ‹ General ☐ 2 Spinal/Epidural

s.9 Mother's health **after delivery** ☐ 1 GOod ☐ z Fair 3 Bad 4 Maternal cleath

Cause of death: ☐

Perot **mcirtem:** ☐ i Yes ☐ i No

5.7 Cestational age at birth ciinical estimate ☐

6 Status of 1. child (Always fill inn)

6.1 Date ofdelivery ☐

6.2 Tjme of delivery ☐

6.3 Sex 1 Male z Female ☐ s Unknown, unspec.

6.4 b irthweight (Gram) ☐

6.5 Length (cm) ☐

6.6 Head circum ☐

6.7 Presentation: ☐ ‹ Cephalic ☐ z Breech ☐ 3 Transverse ☐ ‹ Other

6.8 Status ☐ 1 Live born ☐ 2 Live born transferred to paediatrics dept ☐ * Stillborn ☐ 4 Neonatal death

of oo ☐

6.9 If stillborti: ☐ 1 Dead before labour ☐ z Dead during labour z Unknown, unspec.

If stillborn, also specify: ☐ ‹ Dead before admission ☐ 2 Dead after admission

And: ☐ 1 Fresh ☐ 2 Macerated

Post mortem: 1 Yes ☐ z No

6.10 Apgar score: 1min ☐ **5 min** ☐ **10 min** ☐

If neonatal death: 0 ‹ Died within first 24 hours 2 Died within first week

D te ^e ☐

6.11 Mode of delivery: ☐ 1 Spontaneous ☐ z Vacuum, vaginal ☐ • Forceps, vaginal ☐ • CS elective ☐ s CS others ☐ s Assisted breech ☐ z Destructive operative

Indication when Caesarean section: Primary Secondary ☐

6.1z Failed intervention ☐ 1 Vacuum ☐ z Forceps

6.13 Does the child have any of these conditions? ☐ › Birth defects ☐ z Injuries ☐ • Diseases * HIV Positive ☐

Status on **2x›hild** (For **multiple** births - not for singletons, if more than twins add extra copy of this page)

< 1 Date of delivery ☐

6>2 Time of delivery ☐

.3 Sex • Male ☐ 2 Female ☐ • Unknown, unspec.

6.4 birth wei{ght (gram) ☐

<* Length (cm) ☐

<-< Head circum ☐

6.7 Presentation: ☐ Cephalic ☐ 2 Breech ☐ › Transverse ☐ 4 ther

s.s 3t t • ☐ Live born ☐ • Live born transferred to paediatrics dept ☐ * Stillborn ☐ * Neonatal death

"""•"oo'" ☐

a.e **If stIlJbOF** : ☐ 1 Dead before labour ☐ 2 Dead during labour ☐ s Unknown, unspec.

If stillborn, also specify: ☐ ‹ Dead before admission ☐ 2 Dead after admission

And: ☐ ‹ Fresh ☐ 2 Macerated

Post mortem: i Yes ☐ 2 No

6.10 Apggy **score:** 1rnin ☐ 5 min ☐ **10 min** ☐

If neonatal death: ☐ • Died within first 24 hours ☐ • Died within first week

'e ^oot" ☐

6.11 Mode of **del\V6 »** ☐ ‹ Spontaneous ☐ z Vacuum, vaginal ☐ z Forceps, vaginal < CS elective ☐ s C5 others ☐ • Assisted breech ☐ 7 Destructive operative

Indication when caesarean section: ""* s«•w ☐

s.12 **Failed intervention** **L_I** › Vaccum ☐ z Forceps

6.13 Does the child have any of these conditions? ☐ 1 Birth defects ☐ z Injuries ☐ • Diseases 4 HIV Positive ☐

7 Previous pre anches including abortions in chronolo ical order

CA	C2	C3	C4	C5	C6	C7	C8	C9	C10	C11	C12	C13	C14
bfB§.ñO	Year	Outcome	Months	Birth weight	Sex	Lact.ation m0nths	Delivery where	Attended by	Mode	ANC	Ali Death	Cause of death	Age
1													
2													
3													
4													
5													
6													
7													
8													
9													
10													
11													
12													
13													
14													
15													
16													
17													
18													
19													
C1	C2	C3	C4	C5	C6 *	C7	C8	C9	C1O	C11	C12	C13	C14

C1 Pregnancy number
C2 Year of pregnancy. (Birth or other termination) PP: Present pregnancy
C3 Outcome of pregnancy. (L) Live born, (S) Stillborn, (A) Spontaneous abortion, (I) IhdUced abortion (E) Ectopic. (M) Molar, (T) Twins or other multiples" , (0) Other
C4 Months of gestation at birth or other termination.
c5 Binh weight in grams
C6 Sex: (M) Male, (F) Female, (U) Unknown
C7 Lactation: In months
C8 Delivered where (1) At home, (2) At hospital, (3) At health post, (4) During transport), (5) Other / unknown
c9 Attended by whom: (R) Relative, (N) Nurse, (M} Midwife, (Dt D0ctor, (T) Traditional birth attendant
C10 Mode (S) Spontaneous, (V) Vacuum, (F) Forceps, (C) Cesarean section, (B) Breech, (0) Other, (9) Unknown
C11 Antenatal care: (Y) Yes, (N) No
C12 Child s current status: (A) Alive, (D) Dead
C13 Cause *ol* death: Specify
C14 Age: (1) Less than one week, (2) Less than one month, (3) Le55 than one tear, (4) More than one tear

* Multiple births (TwinS, Triples ...) are filled in on subsequent lines
